# Analysis of Taste Sensitivities in *App* Knock-In Mouse Model of Alzheimer’s Disease

**DOI:** 10.3233/JAD-200284

**Published:** 2020-08-04

**Authors:** Masataka Narukawa, Suzuka Takahashi, Takashi Saito, Takaomi C. Saido, Takumi Misaka

**Affiliations:** a Department of Applied Biological Chemistry, Graduate School of Agricultural and Life Sciences, The University of Tokyo, Tokyo, Japan; b Laboratory for Proteolytic Neuroscience, RIKEN Center for Brain Science, Wako, Saitama, Japan; c Department of Neurocognitive Science, Institute of Brain Science, Nagoya City University Graduate School of Medical Science, Nagoya, Aichi, Japan

**Keywords:** Aging, *App* knock-in mice, donepezil, taste sensitivity

## Abstract

**Background:**

Some studies have reported a decline in taste sensitivities in patients with Alzheimer’s disease. However, the detail remains unknown.

**Objective:**

We investigated the effect of cognitive impairment on taste sensitivity using an *App* knock-in mouse model of Alzheimer’s disease.

**Methods:**

Behavioral assays, a brief access test, and a 48 h two-bottle preference test, to assess taste sensitivities were started from 12 months of age in mice that were confirmed to have impaired cognition.

**Results:**

In the assays, there was no significant difference in taste sensitivities between wild type and *App* knock-in mice. Additionally, no apparent difference was observed in the expression of taste markers in their taste bud cells.

**Conclusion:**

We concluded that cognitive impairment might not greatly affect taste sensitivity.

## INTRODUCTION

Health maintenance of the elderly has become an important social issue with progressively increasing age of the general population in most developed countries. To address the issue, it is imperative to ensure a balanced and nutritional diet in order to maintain their health. A sense of taste is a chemical sensation that mainly commits to the detection of nutrients present in food. Therefore, the maintenance of taste sensation should be important to ensure a balanced nutritional diet for the elderly.

Several reports have indicated that a taste sensitivity gradually changes in an age-dependent manner in humans, although the molecular mechanisms underlying this phenomenon remain unclear. Recently, age-related changes in taste function have reportedly been associated with changes in neuronal circuits [1]. Furthermore, studies have reported a decline in taste sensitivities in patients with Alzheimer’s disease (AD) [2–4]. AD, which is the major form of dementia, is a syndrome caused by a number of progressive illnesses, which affect memory and cognitive capacity in adults aged 65 years and older. However, the effects of brain function, including cognitive function, on taste sensitivity remains unknown.

In this study, in order to understand the possible association between cognitive impairment and taste sensitivity, we employed knock-in (KI) mouse model of AD that endogenously overproduce amyloid-*β* (A*β*) without non-physiological over expression of amyloid-*β* protein precursor (A*β*PP), and measured their taste sensitivity in *App* KI mice. Furthermore, we investigated the effect of an anti-Alzheimer drug on taste sensitivity in wild type mice to validate the participation of the medical therapy in alteration of the taste function.

## METHODS

### Animals

*App^*NL*-*G*-*F*^* mice, which harbor three familial AD mutations (Swedish, Iberian, and Arctic), were used as an *App* knock-in (KI)model. The original line of *App* KI mice was established as C57BL/6J (B6) congenic line (a genetic background strain) by repeated backcrosses [5], and provided by the RIKEN BRC through the National Bio-Resource Project of the MEXT, Japan. B6 mice, which were purchased from CLEA Japan (Tokyo, Japan), were used as wild type (WT) mice. Behavioral experiments were started from 12months of age. Male *App* KI and WT mice were used in this study. All experiments were performed in accordance with protocols approved by The University of Tokyo Animal Care Committee (Approval Number: P17-125).

### Procedure for experiments using App KI mice

Initially, 12-month-old WT (*n* = 6) and *App* KI mice (*n* = 6) performed a Y maze test to assess impairment of working memory. Next, each genotype performed a brief access test, followed by a 48 h two-bottle preference test. After the behavioral experiments, immunohistochemistry experiments were performed with hippocampal and circumvallate papillae (CvP) sections from the same mice, which reached 18 months of age.

### Y maze test

A Y maze had three arms (400 mm deep, 200 mm high, 30 mm wide at the bottom, 100 mm wide at the top) at angles of 120° (Shin factory, Fukuoka, Japan). Mice were placed at the end of one arm and allowed to move freely for 10 min. The frequency of arm entry was counted manually to calculate the total number of entries and the alternation ratio (ratio of actual alternations to maximum alternations, i.e., total number of entries -2).

### Brief access test

The taste sensitivities to tastants were expressed as lick ratios. The lick ratios of the tastants were calculated as follows: number of licks of tastant/number of licks of water. When the lick ratios to attractive taste substances were calculated, the average lick numbers for water during aversive taste test sessions were utilized. To avoid restriction effects, data for mice whose body weight fell below 80% of the *ad libitum* normal free-feeding value were excluded from the analyses. Tastant solutions for the brief access test were (in mM): 0.1–100 citric acid (sour), 0.03–10 denatonium (bitter), 10–1000 NaCl (salty), 1–300 MSG + 0.5 IMP (umami), and 1–300 sucrose (sweet).

The numbers of licks to aversive and attractive taste substances were measured in the first and second weeks, respectively.

Evaluation of aversive taste substances in the first week: Each animal with 23 h water deprivation was placed in a test cage on day 1 of training and given free access to distilled water during a 1 h session. The number of licks per 5 s was measured by a lickometer (Med associates, Fairfax, VT). Days 2–3 were the training session. During this period, the animal was trained to drink distilled water on an interval schedule, consisting of 5 s periods of presentation of distilled water with 30 s intervals. Days 4–5 were the test session. The numbers of licks for denatonium, citric acid, and distilled water by each animal were counted during the first 5 s after the animal’s first lick. After the test session, the mice were rested.

Evaluation of attractive taste substances in the second week: Each animal was limited in its water intake to the average intake of water during the normal breeding period. Days 8–11 were the training session. On days 12–14, the numbers of licks for sucrose, MSG + IMP, and NaCl by each animal were counted during the first 5 s after the animal’s first lick.

### Forty-eight-hour two-bottle preference test

The mice were given 48 h of access to two bottles, one containing deionized water and the other containing a tastant solution. After 24 h, the bottle positions were switched to avoid positional effects. The preference ratio for the tastants was calculated as follows: tastant intake/total fluid intake (tastant intake + water intake). The tastant solutions for the two-bottle preference test were (in mM): 1–100 citric acid, 0.03–3 denatonium, 10–500 NaCl, 0.1–10 MSG + 0.5 IMP, and 10–100 Sucrose. The solutions for each tastant were presented in an ascending series of concentrations.

### Immunohistochemistry

For brain and CvP preparations from WT and *App* KI mice, mice were sacrificed by an overdose of intraperitoneal sodium pentobarbital and transcardially perfused with ice-cold phosphate-buffered saline (PBS), followed by treatment with 4% paraformaldehyde (PFA) in PBS. Brain and CvP were dissected, post-fixed in 4% PFA/PBS at 4°C overnight, cryoprotected in a series of graded concentrations of 20% sucrose/PBS at 4°C, and frozen in an O.C.T. compound (Sakura Finetek, Tokyo, Japan). The frozen blocks containing brain and/or CvP were stored at –80°C until use. The brains were sliced coronally into 25*μ*m free-floating sections using a retoratome (REM-710; Yamato Kohki Industrial, Saitama, Japan) and stored in PBS with 0.02% NaN_3_ at 4°C until further use. The CvP were sectioned at 10*μ*m with a cryostat (Cryostar NX70; Thermo Scientific, Waltham, MA). The sections were mounted onto MAS-coated glass slides (Matsunami Glass, Osaka, Japan) and stored at – 80°C until further use.

For brain, sections were incubated with 70% formic acid at 5 min for antigen retrieval. After washing with PBS containing 0.1% Triton X-100 (PBS-T), the sections were blocked in Blocking One (NacalaiTesque, Kyoto, Japan), and then incubated overnight at 4°C with mouse anti-A*β* 82E1 (1 : 200; IBL, Gunma, Japan) and rabbit anti-GFAP (1 : 500; SHIMA Laboratories, Tokyo, Japan). After washes with PBS-T, the sections were then incubated for 1 h with Alexa Fluor 488 donkey anti-mouse IgG (1 : 500; Thermo Fisher Scientific) and Alexa Fluor 555 donkey anti-rabbit IgG (1 : 500; Thermo Fisher Scientific). After three washes with PBS-T, the sections were incubated with DAPI (1*μ*g/ml) at 5 min and mounted with Fluoromount (Diagnostic Biosystems, Pleasanton, CA).

For the CvP, the sections were washed with PBS and incubated in antigen retrieval solution for 20 min at 80°C (Dako Target Retrieval Solution, pH 9, Agilent Technologies, Santa Clara, CA). The slides were then blocked with PBS containing Blocking One and incubated overnight at 4°C with rabbit anti-Gustducin (1 : 500, Santa Cruz Biotechnology, Dallas, TX) and goat anti-Kcnq1 (1 : 1000, Santa Cruz Biotechnology), or rabbit anti-Plc*β*2 (1 : 500, Santa Cruz Biotechnology) and goat anti-Car4 antibodies (1 : 500; R&D systems, Minneapolis, MN). The sections were then washed with PBS and incubated with Alexa Fluor 488 donkey anti-rabbit IgG (1 : 500, Thermo Fisher Scientific) and Alexa Fluor 555 donkey anti-goat IgG (1 : 500, Thermo Fisher Scientific) for 1 h at room temperature. The sections were then rinsed with PBS and mounted with Fluoromount.

The fluorescent and differential interference contrast (DIC) images were taken with a BX51 microscope equipped with a DP71 digital camera (Olympus, Japan). Brightness and contrast of fluorescence images were adjusted and each image was merged using Adobe Photoshop Elements 14 (Adobe Systems, San Jose, CA).

### Donepezil treatment

The effect of donepezil on taste sensitivity was determined by the brief access test. Eight-week-old male B6 mice were intraperitoneally administered with saline (*n* = 6) or donepezil hydrochloride (1 mg/kg body weight, *n* = 6, NakalaiTesque, Kyoto, Japan) once daily. The brief access test protocol followed the experimental method with *App* KI mice. The test period was approximately 2 weeks long. After 3 days of training sessions, the numbers of licks for denatonium and citric acid were counted on days 4 and 5, respectively. The mice completed new training sessions on days 8–11. The numbers of licks for sucrose, MSG + IMP, and NaCl were counted on days 12–14. Donepezil was administered daily after each training or assessment session. The administration period lasted from the beginning of training to the end of testing. Donepezil was also administered during the rest period (days 6 and 7).

### Statistical analysis

The results were expressed as mean±standard error of the mean. All statistical analyses were performed using Graph Pad Prism 6 software (GraphPad Software, CA, USA). Differences were evaluated using Welch’s *t*-test or two-way repeated-measures analysis of variance (ANOVA). For all analyses, *p* < 0.05 were considered statistically significant.

## RESULTS

**Fig. 1:**
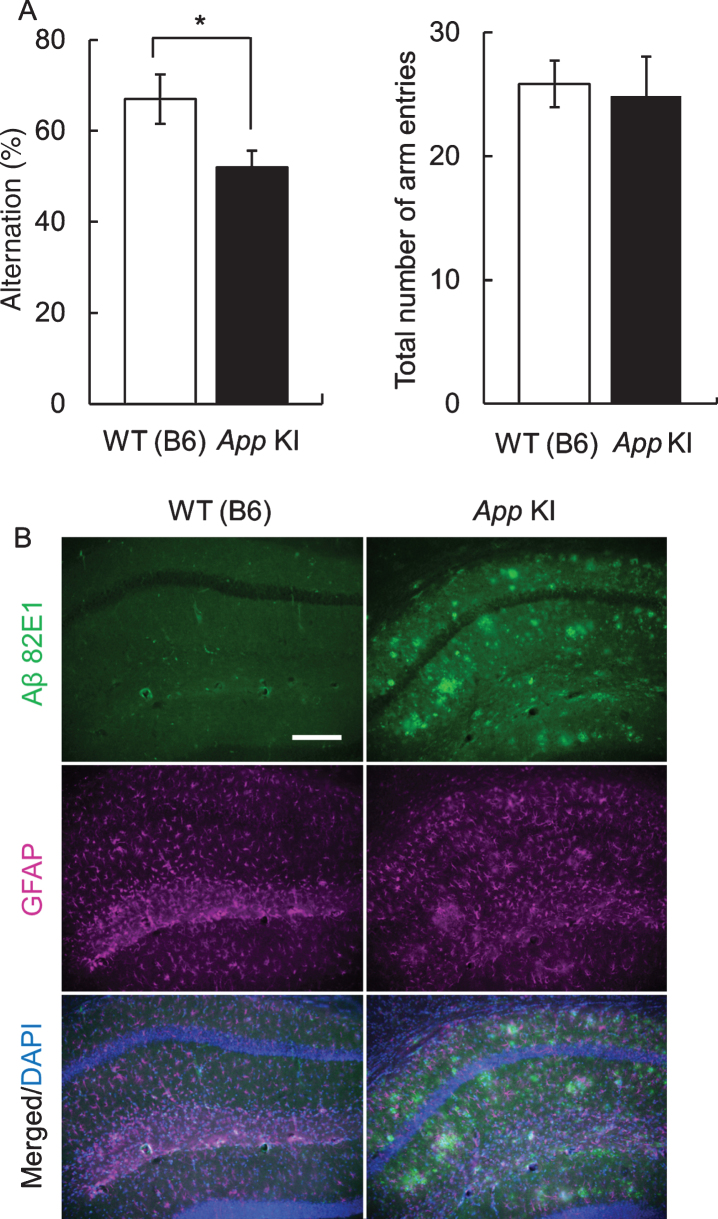
Cognitive impairment and neuropathology of *App* knock-in (KI) mice. A) Cognitive impairment in *App* KI mice. The Y-maze test was performed using 12-month-old wild type (WT) and *App* KI mice. *p < 0.05 (Welch’s *t*-test, *n* = 6). B) A*β* deposition of WT and *App* KI mice. The hippocampus samples wereprepared from 18-month-old mice that had completed the behavioral experiments. Representative images of the hippocampal regions from coronal brain sections immuno stained with anti-A*β* 82E1 (green) and anti-GFAP (magenta) are shown (blue in merged images indicated DAPI staining). Scale bar is 50*μ*m.

First, we confirmed the impairment of cognitive function by the Y maze test. In 12-month-old mice, there was no difference in the total number of arm entries between WT and *App* KI mice. However, the alteration ratio in *App* KI mice was significantly decreased in comparison with that in WT mice (Fig. 1A). In addition, A*β* deposition was also confirmed in the hippocampus of 18-month-old *App* KI mice, which we obtained from the mice at the end of behavioral experiments, but was not observed in WT mice (Fig. 1B).

**Fig. 2:**
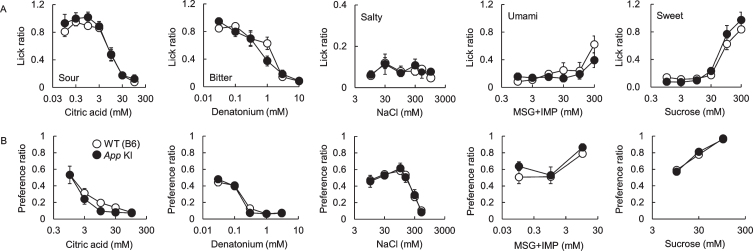
Comparison of taste sensitivities for basic tastes in wild type (WT) and *App* knock-in (KI) mice. The lick (A) and preference (B) ratios for citric acid, denatonium, NaCl, MSG + 0.5 mM IMP, and sucrose are shown. White and black circles indicate WT and *App* KI mice, respectively.

**Fig. 3:**
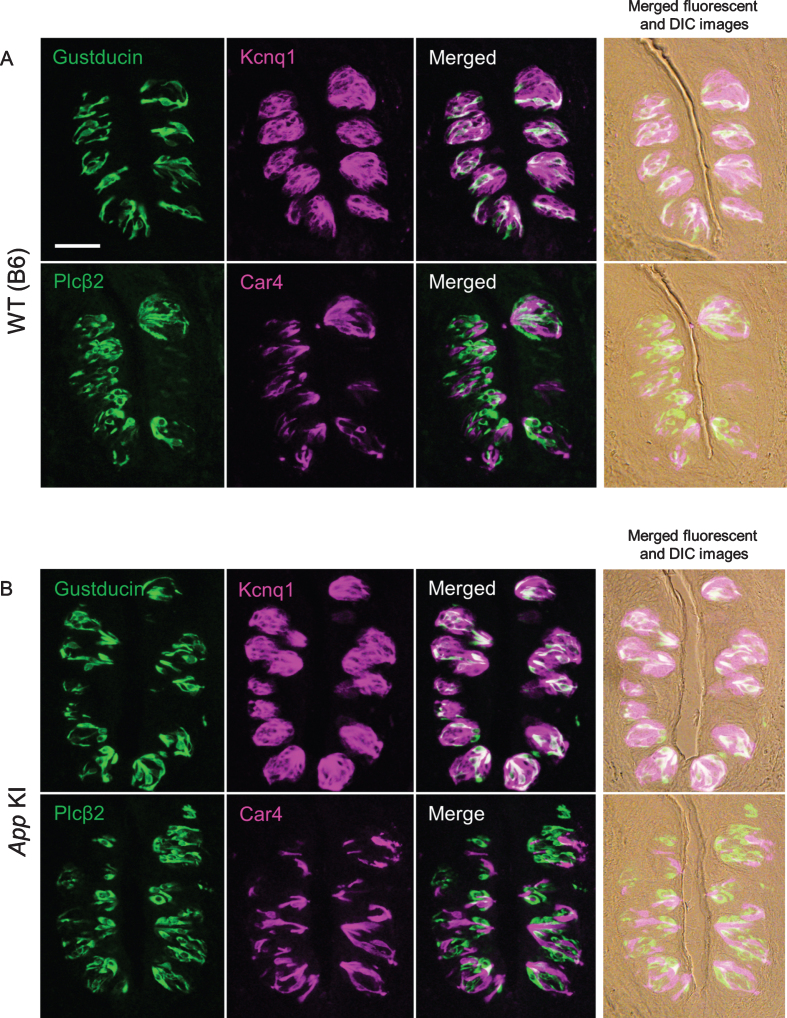
Expression of taste marker molecules in the circumvallate papillae of wild type (WT) (A) and *App* knock-in (KI) mice (B). Representative double-fluorescence immunostaining images are shown. The immunostaining was examined using antibodies against Gustducin (green) and Kcnq1 (magenta), and Plc*β*2 (green) and Car4 (magenta). Right column shows merged fluorescence and differential interference contrast (DIC) images. Scale bar is 50*μ*m.

After we confirmed the impairment of cognitive function, taste sensitivities to five basic tastes (sour, bitter, salty, umami, and sweet) were measured by the brief access test and 48 h two bottle preference test. There was no significant difference in taste responses between WT and *App* KI mice in both tests (Fig. 2A for the brief access test, citric acid: F_ (1,70) _ = 1.95, *p* = 0.17; denatonium: F_ (1,60) _ = 0.73, *p* = 0.40; NaCl: F_ (1,60) _ = 0.17, *p* = 0.69; MSG + IMP: F_ (1,60) _ = 1.62, *p* = 0.21; sucrose: F_ (1,60) _ = 0.89, *p* = 0.35, Fig. 2B for the two bottle test, citric acid: F_ (1,50) _ = 2.53, *p* = 0.12; denatonium: F_ (1,50) _ = 0.03, *p* = 0.78; NaCl: F_ (1,60) _ = 0.03, *p* = 0.85; MSG + IMP: F_ (1,30) _ = 2.17, *p* = 0.15; sucrose: F_ (1,30) _ = 0.01, *p* = 0.94). We also observed the expression of representative taste-related proteins (i.e., the type II taste cell markers Gustducin and Plc*β*2, the type III taste cell marker Car4, and the taste bud cell marker Kcnq1) in the taste buds of the CvP. Immuno reactivity to the relevant antibodies was observed in mice of both genotypes with no apparent differences in protein expression patterns (Fig. 3). We observed no apparent differences in CvP morphologies between the genotypes (Fig. 3).These results suggest that the cognitive impairment observed in AD may not greatly affect taste sensitivity.

**Fig. 4:**
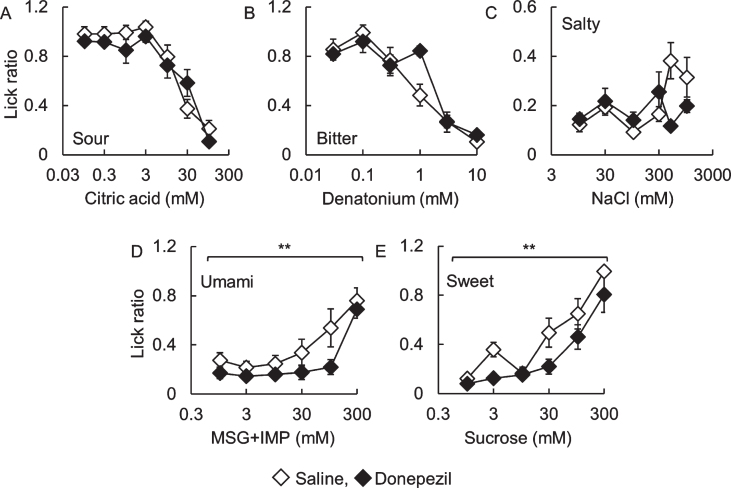
Lick ratios for basic tastes in donepezil-administered C57BL/6J mice.The lick ratios for (A) citric acid, (B) denatonium, (C) NaCl, (D) MSG + 0.5 mM IMP, and (E) sucrose are shown. White and black diamonds indicate saline- and donepezil-administered mice, respectively. **p < 0.01 (two-way repeated-measures ANOVA, *n* = 6).

Next, we investigated the effect of donepezil administration, the major compound for the treatment of AD, on taste sensitivity in B6 mice (Fig. 4). There were no significant differences in lick ratios toward sour, bitter, and salty solutions between saline- and donepezil-administered mice (Fig. 4A to C, citric acid: F_ (1,70) _ = 1.35, *p* = 0.25; denatonium: F_ (1,60) _ = 1.12, *p* = 0.29; NaCl: F_ (1,60) _ = 1.45, *p* = 0.23). However, lick ratios to sweet and umami solutions in donepezil-administered mice were significantly lower than those in saline-administered mice (Fig. 4D and E, MSG + IMP: F_ (1,60) _ = 8.91, *p* < 0.01; sucrose: F_ (1,60) _ = 12.0, *p* < 0.01). This result suggests that donepezil administration induces a change in taste sensitivity. Meanwhile, there were no apparent differences in the expression patterns of type II taste cell markers IP_3_R3, type III taste cell marker Ncam, and Kcnq1 between both mice (data not shown).

## DISCUSSION

We have previously compared the peripheral taste detection systems between young and old mice to investigate the contributing factors associated with aging-dependent changes in taste sensitivity. Although an aging-associated change in taste sensitivities was noted, aging-related degradation of peripheral taste organs, such as expression levels of taste-related molecules and turnover rate of taste bud cells, was not confirmed [6, 7]. Therefore, we concluded that changes in taste sensitivity due to aging are caused by factors other than those responsible for the deterioration of taste detection systems in the oral cavity. A recent study has reported that age-related changes in taste function are related to alterations in neuronal circuits [1]. Additionally, it has been reported that taste threshold could decrease in patients with AD [2–4]. Therefore, functional changes in the central nervous system, such as cognitive functional decline accompanying with aging, may be considered as a candidate to elucidate the underlying mechanisms in aging dependent changes in taste sensitivities. Therefore, using the *App* KI mouse model of AD, we here investigated the impact of cognitive impairment on taste sensitivity.

In this study, taste sensitivity was examined by two behavioral assays, the brief access test and 48 hours two-bottle preference test, which reflect different characteristics of taste response, using 12-month-old mice that were confirmed to have cognitive impairment (Fig. 2). The two-bottle preference test is a conventional method for determining taste preference. Taste preference reflects orosensory stimulation as well as post-ingestive effects [8]. The brief access test is based on preferential licking behavior toward a taste solution, and quantifies taste sensitivity and preference by measuring the initial licking rate. As the initial licking rate is measured within one minute, post-ingestive effects are completely excluded [8]. However, the mice have restrictions on eating and drinking. As such, we performed both tests in the present study. Interestingly, we found no significant difference in taste sensitivities between WT and *App* KI mice in both behavioral assays (Fig. 2). Furthermore, no apparent difference was observed in the expression of representative taste markers in the CvP and the morphology of CvP between the genotypes (Fig. 3). These results suggest that cognitive impairment may not affect taste sensitivity in AD. We recently reported that intake of *α*-glycerophosphocholine, a precursor of the neurotransmitter acetylcholine, improved aging-related decline in gene expression levels of long-term potentiation in the hippocampus but not taste sensitivity [9]. This result is in line with the results found here.

What does cause decline in taste sensitivity observed in AD patients? It is known that drug intake induces taste disorders [10]; therefore, we focused on representative anti-Alzheimer drug donepezil. Donepezil is the major compound for the treatment of AD in more than 50 countries, and is a cholinesterase inhibitor [11]. We investigated the effect of donepezil administration on taste sensitivity in young B6 mice. As a result, donepezil administration induced a decline in taste response for umami and sweet tastes (Fig. 4). Thus, medication may be one of the reasons for the observed decline in taste sensitivity in AD patients. In fact, Ogawa et al. reported that all AD volunteers, which investigated taste threshold in their study, took anti-Alzheimer drugs [4]. On the other hand, in AD patients, although a decline in taste sensitivity, measured by the filter paper disc method, was observed, no difference was found in the electrogustometric thresholds [4]. This suggests that impairment of the peripheral taste system may not cause the decline of taste thresholds in AD patients. We did not observe the apparent differences in the expression of representative taste markers in the CvP and the morphology of CvP in donepezil-administered mice (data not shown).

We observed different patterns of lick ratios, especially umami and sweet, between *App* KI (Fig. 2A) and donepezil treatment mice (Fig. 4). We have observed that mice show different taste behaviors depending on their life stage [6, 7]. This dependency on assay timing means that comparing individual lick values from different experimental conditions is not appropriate. The reason why licking behaviors differed between the *App* KI and donepezil experiments could be the difference in the ages and assessment timings.

Donepezil administration in mice affected licking behaviors to umami and sweet (Fig. 4), while changes of taste thresholds toward other than umami and sweet have observed in AD patients [3, 4]. We intend to further investigate the mechanism of taste sensitivity change by drug intake (e.g., amount and period of drug intake) in a future study.
